# Doubled haploid technology for line development in maize: technical advances and prospects

**DOI:** 10.1007/s00122-019-03433-x

**Published:** 2019-09-25

**Authors:** Vijay Chaikam, Willem Molenaar, Albrecht E. Melchinger, Prasanna M. Boddupalli

**Affiliations:** 1 International Maize and Wheat Improvement Center (CIMMYT), ICRAF campus, UN Avenue, Gigiri, P.O. Box 1041, Nairobi, 00621 Kenya; 2 Institute of Plant Breeding, Seed Science and Population Genetics, University of Hohenheim, 70593 Stuttgart, Germany

## Abstract

**Key Message:**

Increased efficiencies achieved in different steps of DH line production offer greater benefits to maize breeding programs.

**Abstract:**

Doubled haploid (DH) technology has become an integral part of many commercial maize breeding programs as DH lines offer several economic, logistic and genetic benefits over conventional inbred lines. Further, new advances in DH technology continue to improve the efficiency of DH line development and fuel its increased adoption in breeding programs worldwide. The established method for maize DH production covered in this review involves in vivo induction of maternal haploids by a male haploid inducer genotype, identification of haploids from diploids at the seed or seedling stage, chromosome doubling of haploid (*D*_0_) seedlings and finally, selfing of fertile *D*_0_ plants. Development of haploid inducers with high haploid induction rates and adaptation to different target environments have facilitated increased adoption of DH technology in the tropics. New marker systems for haploid identification, such as the red root marker and high oil marker, are being increasingly integrated into new haploid inducers and have the potential to make DH technology accessible in germplasm such as some Flint, landrace, or tropical material, where the standard *R1*-*nj* marker is inhibited. Automation holds great promise to further reduce the cost and time in haploid identification. Increasing success rates in chromosome doubling protocols and/or reducing environmental and human toxicity of chromosome doubling protocols, including research on genetic improvement in spontaneous chromosome doubling, have the potential to greatly reduce the production costs per DH line.

## Introduction

Production of homozygous inbred lines as parental lines of hybrids or synthetic varieties is an important component of maize breeding programs. This was recognized as early as 1908 by Shull (1908), who proposed that the essential task of the maize breeder is to find the best hybrid combination of parents in a maize population to generate seed corn that shall produce the record crop. During the twentieth century, development of inbred lines in maize relied almost exclusively on six to eight generations of recurrent selfing and selection to reach the desired level of homozygosity (Hallauer et al. 2010). When the extensive field trials for variety registration are included, it usually takes up to 11–13 years from the time of the initial crosses to the release of new cultivars onto the market. As a result, maize breeders and geneticists have been eager to adopt methods to speed up the process of inbred line production.

In the last two to three decades, doubled haploid (DH) technology has emerged as an efficient alternative to the traditional method of inbred line development. The technology essentially samples the segregating gametes of the source germplasm, usually a biparental cross or a population, and produces completely homozygous lines in one step. Both in vitro and in vivo methods can be used to develop maize DH lines. However, in vivo methods have proved to be more reliable and efficient in large-scale production of DH lines and hence are commonly used in maize.

In vivo DH technology has been adopted by many commercial maize breeding programs in Europe, North America, and China (Molenaar and Melchinger 2019). Although application of DH technology in tropical breeding programs has been slow (Kebede et al. 2011; Prasanna 2012), it has now gained a foothold due to intensive efforts by International Maize and Wheat Improvement Center (CIMMYT), in collaboration with the University of Hohenheim, making the technology accessible to both public and private sector organizations. In recent years, improved maize hybrids with DH lines as parental lines have been released in Africa (Beyene et al. 2017; Chaikam et al. 2018). This review focuses on the advantages and applications of in vivo production of DH lines in maize breeding, and recent advances in the four technical steps involved in DH line development.

## Advantages of DH lines in maize breeding

DH technology offers the fastest and most efficient route to produce completely homozygous lines for maize breeding programs. It allows the development of homozygous inbred lines in a single year, compared to three to 4 years of inbreeding with the conventional recurrent selfing method, when using off season nurseries. Residual heterozygosity in conventional inbred lines can sometimes delay plant variety registration, while complete homozygosity of DH lines makes them very amenable for variety registration/protection because they comply with Distinctness, Uniformity and Stability (DUS) criteria (Röber et al. 2005). Considering this, the most significant advantage of DH technology to maize cultivar development has been the reduced time to commercialization (Seitz 2005; Bordes et al. 2006). In a comparison among DH, pedigree and single seed descent methods of inbred line development under different testing regimes like conventional 3-year phenotyping, accelerated 2-year phenotyping and single year phenotyping with genomic selection, Atlin et al. (2017) showed a reduction in the overall breeding cycle time of at least 1 year when using DH compared to single seed descent and 2 years compared to pedigree methods.

Conventionally, during inbreeding breeders develop thousands of segregating plants/families in early generations and hundreds of inbreds with various degrees of homozygosity in advanced generations from each population. When using DH technology, desired numbers of finished inbred lines can be attained at once, eliminating the need for handling larger numbers of breeding materials from different generations of inbreeding. Consequently, when using DH technology, logistics like shipping the seed, managing inventories, planting nurseries, selfing and maintaining lines become much simpler (Röber et al. 2005; Prasanna 2012). In the long term, simplified logistics lead to significant cost savings for breeding programs (Jumbo et al. 2011).

Use of DH lines offers opportunities for improving selection gain. Complete homozygosity of DH lines allows more accurate phenotyping over multiple locations and years compared to families in early selfing generations (e.g., F_3_ or F_4_) (Foiada et al. 2015; Yan et al. 2017). In addition, the relatively high genetic variance in DH lines increases response to selection (Melchinger et al. 2005; Bordes et al. 2007; Gallais and Bordes 2007; Mayor and Bernardo 2009) by increasing heritabilities for various traits during per se and test cross evaluation. Higher genetic gain per year is realized in commercial maize breeding programs using DH lines because of reduced time in inbreeding and increased genetic variance, which requires special attention for optimum allocation of resources (Longin et al. 2006, 2007a, b; Mi et al. 2011; Sleper and Bernardo 2016; Wegenast et al. 2008, 2009, 2010).

Use of DH lines in combination with molecular markers offers further opportunities for increasing selection gain. Segregation ratios of 1:1 for both dominant and codominant markers make DH lines ideal for marker applications (Yan et al. 2017). DH methods in combination with marker-assisted selection (MAS) allow quicker and more efficient fixation of favorable alleles. Thus, combining molecular markers and DH technology is a very powerful tool for target gene fixation and stacking of genes (Melchinger et al. 2011) because the frequency of desirable homozygous genotypes decreases at a lower rate in DH lines than in backcross genotypes. This is because for “g” independently segregating genes, the expected frequency of favorable homozygotes is 0.25 ^g^ in BCnF_2_ populations vs 0.5 ^g^ in DH lines (Lübberstedt and Frei 2012). As a result, use of DH lines saves costs in sample collection, DNA extraction, genotyping, and data analysis. Complete homozygosity of DH lines offers a higher phenotype to genotype correlation, thereby facilitating better estimation of quantitative trait loci (QTL) effects in marker-trait association studies (Hyne et al. 1995). In addition, DH lines are expected to improve the selection response compared to F_2_ populations, as shown by simulation studies in marker-assisted recurrent selection and genome-wide selection, when dealing with complex traits of low heritability controlled by many QTLs (Mayor and Bernardo 2009).

DH technology also enables more effective access to the genetic diversity from landraces and open-pollinated varieties (OPVs). A high genetic load of deleterious alleles and high heterogeneity prevent the use of landraces and OPVs in hybrid maize breeding (Wilde et al. 2010; Melchinger et al. 2018). Deleterious alleles from such germplasm are readily expressed in the haploid stage and can then be purged through natural or artificial selection. Hence, DH technology is a highly effective tool to access the genetic diversity present in allogamous landraces and to expand the genetic diversity of elite germplasm (Wilde et al. 2010; Strigens et al. 2013; Böhm et al. 2017; Brauner et al. 2019; Hölker et al. 2019). The DH lines from landraces and OPVs can be evaluated in replicated trials with high precision, which is not possible when using landraces and OPVs due to their high heterogeneity (Strigens et al. 2013). Moreover, genomic selection can be applied successfully to DH lines produced from biparental populations and landraces (Brauner et al. 2018; Han et al. 2018). Random samples of DH lines derived from landraces were proposed to be ideal for association mapping due to low population structure and quick decay of linkage disequilibrium (Strigens et al. 2013; Melchinger et al. 2018).

## Production of maize DH lines

The production of maize DH lines involves the following four steps: (1) induction of haploids; (2) identification of haploids at seed or seedling stage; (3) chromosome doubling in the haploids; and (4) selfing the fertile doubled haploid plants to produce seed for DH lines.

### Haploid induction

Both in vitro and in vivo methods can be used to obtain maize haploids; however, in vitro methods have shown little promise to reliably produce the large numbers of DH lines required by maize breeding programs, and as a result are currently not being used (Chaikam 2012; Liu et al. 2016). In contrast, in vivo haploid induction paved the way for large-scale production of DH lines and almost all DH production pipelines in the maize breeding programs of multinational companies are now based on in vivo haploid induction. Discovery of the natural occurrence of haploid maize plants between the 1920s and 1960s, albeit at low frequencies (~ 0.1%), laid the foundation for in vivo DH production (Chase 1969). Such spontaneously occurring haploids were used to produce some of the commercial inbred lines and hybrids in the USA and Europe during the 1950s and 1960s. However, low frequency of naturally occurring haploids coupled with inefficiencies in later production steps made it difficult to produce DH lines on a large scale. A major breakthrough came when certain maize genotypes, called “haploid inducers,” were identified which increased the frequency of haploid production compared to normal maize (Coe 1959). Haploid inducers are classified as paternal or maternal inducers on the basis of the genetic constitution of the resulting haploids. Paternal and maternal haploids carry a haploid genome from the male and female parent, respectively. Paternal haploid inducers are used as female parents and the source germplasm from which haploids are desired is used as the male parent. Paternal haploid induction results from mutation in the *ig1 (indeterminate gametophyte*) gene (Kermicle 1969, 1971; Evans 2007). Up to this point, *ig1*-based paternal haploid induction has not been commonly used in maize breeding programs because it induces haploids at low frequency (~1–2%) (Pollacsek 1992; Kermicle 1994) and the cytoplasmic constitution of the resulting haploids is different from the source germplasm (Kermicle 1973). However, derivation of paternal haploids can be particularly useful for conversion of inbred lines to their isogenic cytoplasmic male sterile form (Geiger 2009; Weber 2014).

**Fig. 1:**
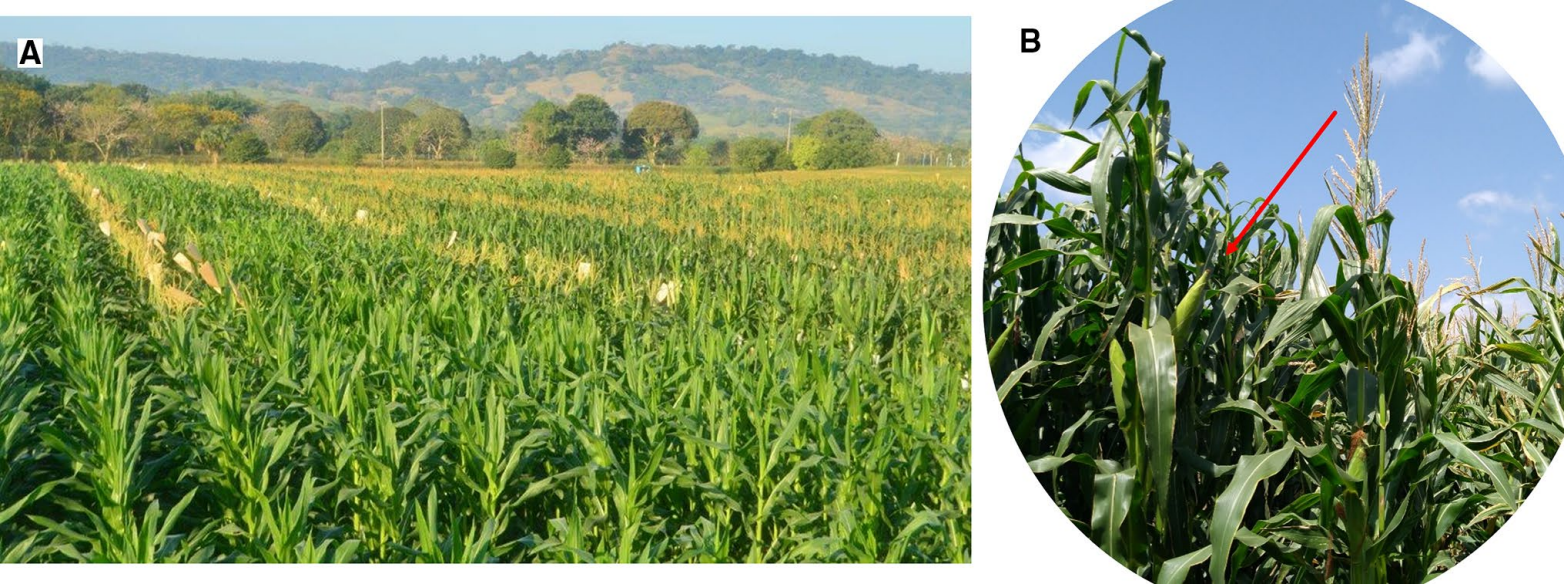
Haploid induction based on in vivo maternal haploid inducers. **a** A haploid induction nursery where plants with tassels are haploid inducers, and the detasseled plants are from different source germplasm. **b** Pollination of the ears of source germplasm with the haploid inducer

In maternal haploid induction, the source germplasm is used as the female parent and the haploid inducer is used as the male parent (Fig. 1). The resulting haploids inherit both the cytoplasmic and nuclear genome from the female parent, thereby making maternal haploid induction more appealing for maize breeding programs. The influence of paternal genotypes on haploid induction was elaborated by Chase (1949a) and led to the search for new pollinator stocks with high haploid induction frequency. The first inducer recorded, called “Stock 6,” produced maternal haploids at a frequency of 1–3%, which was the highest haploid induction rate (HIR) at the time (Coe 1959). Further efforts to improve the HIR and adaptation to different environments led to development of several Stock 6-based inducers with high HIR (Sarkar et al. 1972; Aman and Sarkar 1978; Zavalishina and Tyrnov 1984; Liu and Song 2000; Shatskaya 2010). Using Stock 6-derived haploid inducers, several highly effective inducers with high HIR and good agronomic performance have been developed during the last two decades. These inducers include UH400 (https://plant-breeding.uni-hohenheim.de/84531#jfmulticontent_c167370-2), RWS (Röber et al. 2005), MHI (Chalyk 1999), and PHI (Rotarenco et al. 2010), which are all adapted to temperate environments, and these have laid the foundation for large-scale production of DH lines in commercial maize breeding programs. These inducers have HIR of 6–15%.

Before 2012, haploid inducers with good adaptation and a high HIR were not available for tropical environments, especially in the public domain. CIMMYT and the University of Hohenheim developed the first-generation Tropically Adapted Inducer Lines (TAILs), which have superior agronomic performance compared to temperate inducers as well as an acceptable HIR of 6–9% (Prigge et al. 2012a; Chaikam et al. 2016). Recognizing the potential for further improvement in the HIR and agronomic performance of TAILs, CIMMYT developed second-generation TAILs that have much higher HIR (9–14%) and better agronomic performance compared to the first-generation TAILs (Chaikam et al. 2018).

Several reproductive abnormalities like kernel abortion, segregation distortion, heterofertilization and twin plants are often noted to be associated with maternal haploid induction. Higher rates of endosperm and embryo abortion leading to formation of defective kernels is observed when using inducer pollen for selfing and crossing compared to when using non-inducer pollen (Prigge et al. 2012b; Xu et al. 2013; Qiu et al. 2014; Li et al. 2017; Nair et al. 2017; Kelliher et al. 2017; Chaikam et al. 2018; Tian et al. 2018). Generally, inducers with a high HIR have also high kernel abortion (Li et al. 2017; Chaikam et al. 2018); however, there is no tight correlation especially between endosperm abortion and the HIR (Chaikam et al. 2018). It has also been shown that almost all the aborted seeds and haploid seeds contain the inducer genotype at a locus critical for haploid induction (discussed below) (Xu et al. 2013). Hence, it can be inferred that the same genetic mechanisms are responsible for haploid induction and kernel abortion (Xu et al. 2013). The development stage at which kernel abortion happens has not yet been clearly established, and it could be during either fertilization or post-fertilization due to failure of double fertilization or suspension of zygote or endosperm development.

In crosses involving non-inducer and inducer genotypes, a higher than expected percentage of non-inducer plants was recorded in the progenies, indicating segregation distortion against the inducer genotypes (Barret et al. 2008; Prigge et al. 2012b; Dong et al. 2013, 2014; Nair et al. 2017; Chaikam et al. 2018). It was shown that a high HIR in a maternal haploid inducer is associated with high levels of segregation distortion and both gametophytic and zygotic selection contributes to segregation distortion (Xu et al. 2013). Segregation distortion may be the result of natural selection disfavoring the haploid induction trait because a high proportion of haploids in the progenies result in fitness disadvantages due to their high rates of sterility (Prigge et al. 2012b). Abortion of kernels with the inducer genotype may play a critical role in the segregation distortion associated with haploid induction.

Another reproductive anomaly known to be associated with haploid induction is a high frequency of heterofertilization where the egg cell and central cell are fertilized by sperm cells from different pollen grains (Sarkar and Coe 1966; Rotarenco and Eder 2003; Liu et al. 2017a; Tian et al. 2018). Heterofertilization is usually caused by delayed fertilization and is highly influenced by pollen competitive ability (Liu et al. 2017a). Heterofertilization suggests a fertilization recovery mechanism to compensate for initial failure of double fertilization through acceptance of a second pollen tube (Dresselhaus and Sprunck 2012; Liu et al. 2017a).

In induction crosses involving maternal haploid inducers, a high frequency of occurrence of twin plants was also noticed (Sarkar and Coe 1966; Chase 1969). The ploidy status of twins could be diploid-diploid, diploid-haploid and haploid-haploid (Sarkar and Coe 1966). It was shown that the HIR of an inducer is correlated with the rate of twin embryo seeds and that most twins develop from cleavage of the developing embryo (Liu et al. 2018).

#### Genetics of haploid induction

Haploid induction is a quantitative trait controlled by a few genes (Lashermes and Beckert 1988). Several QTL mapping studies with biparental populations between inducers and non-inducers have led to identification of at least eight QTLs (Barret et al. 2008; Deimling et al. 1997; Prigge et al. 2012b). A first mapping study reported by Deimling et al. (1997) indicated that two QTLs on chromosomes 1 and 2 explained 17.9% of the phenotypic variance. Barret et al. (2008) used a segregation distortion method to map QTLs conditioning haploid induction and identified a major QTL on chromosome 1.

A major study employing multiple mapping populations identified the QTLs *qhir1* on chromosome 1 (bin 1.04) and *qhir8* on chromosome 9 (bin 9.01), explaining 66% and 20% of the genotypic variance for HIR, respectively, along with several other minor QTLs (Prigge et al. 2012b). All the above-mentioned QTL mapping studies pointed to a region in or close to the physical intervals of *qhir1*, and this region was thus considered mandatory for conditioning haploid induction. Later, *qhir1* was fine mapped to a region of 243 kb in length (Dong et al. 2013). A genome-wide association study (GWAS) was also conducted using 53 inducers and 1482 non-inducers (Hu et al. 2016), which separated *qhir1* into two regions, *qhir11* and *qhir12*, with *qhir11* containing the region fine mapped by Dong et al. (2013). However, evaluation of the HIR of genotypes containing sub-regions *qhir11* and *qhir12* revealed that only *qhir11* had a significant effect on haploid induction (Nair et al. 2017). Within the *qhir11* region, a phospholipase A encoding gene was identified as being responsible for haploid induction by three research groups independently and named MATRILINEAL (MTL) (Kelliher et al. 2017), NOT LIKE DAD (NLD) (Gilles et al. 2017), and ZmPLA1 (Liu et al. 2017b). Here after, this gene is referred as MTL in this article. Validation of the effect of the mutant allele of this gene by different approaches including backcrossing, and TALEN or CRISPR/Cas9 technology revealed different induction rates (0.5–12.5%) conditioned by the mutant allele. These variable HIRs can be attributed to the quantitative nature of maternal haploid induction and effects of other genes in the genetic background. This was recently confirmed when a second major QTL, *qhir8*, was dissected and the nucleotide sequence variation influencing the haploid induction was identified in the ZmDMP gene that encodes a DUF679 domain membrane protein (Zhong et al. 2019). Knockout of the wildtype ZmDMP resulted in a nominal HIR of 0.1–0.3% in the absence of mutation in MTL gene. But when accompanied with the mutation in the MTL gene, it resulted in 5–6-fold increase in HIR. Together, these results suggest that mutation in the MTL gene is critical for haploid induction and the HIR conditioned by this mutation can be positively influenced by mutation in the ZmDMP gene. The mechanism by which the MTL gene conditions haploid induction and how the interaction of this gene with ZmDMP increases the HIR is yet to be deciphered.

In addition to the haploid inducer, a significant influence of source germplasm on HIR has been reported, with a preponderance of general combining ability effects (Kebede et al. 2011). Prigge et al. (2011) also found significant genetic variation for induction rates across elite tropical germplasm and measured higher induction rates in some single crosses and landraces than in open-pollinated varieties. Significant variation in the maternal genotypes for haploid inducibility was also confirmed in other studies (Wu et al. 2014; De La Fuente et al. 2018). Wu et al. (2014) detected two QTLs for the HIR in the source germplasm, named *qmhir1* and *qmhir2*, and these explained 14.7 and 8.4% of the total genetic variance of the trait, respectively. At CIMMYT, an analysis of the HIR in induction crosses from 672 tropical elite inbred lines revealed significant genetic variation for haploid inducibility (Nair et al. personal communication). A GWAS conducted on these lines for the influence of the maternal parent on the HIR led to identification of several genomic regions that affect haploid induction. Some inbred lines examined in this study have shown more than double the HIR of the mean HIR of all the lines. Such lines could be used to improve the haploid induction capability of elite germplasm.

#### Mechanism of in vivo maternal haploid induction

The mechanism/s underlying maternal haploid induction have not yet been elucidated. It has been clearly established that pollen in maternal haploid inducers is responsible for inducing haploids (Coe 1959). Analysis of thousands of pollen grains from inducer Stock 6 has revealed that all of them are normal with three nuclei, thereby ruling out the possibility of monospermy as a cause of haploid induction (Sarkar and Coe 1966). Also, the endosperm in haploid seeds was determined to be triploid rather than tetraploid, ruling out the possibility of two haploid sperm cells or a single diploid cell fertilizing the central cell (Chase 1964a; Sarkar and Coe 1966). In vitro-germinated inducer pollen grains have generated two-pollen tubes instead of one at a higher frequency than non-inducers (Pogna and Marzetti 1977). Dual pollination experiments with inducer pollen followed by non-inducer pollen has revealed that viable inducer pollen has poor competitive ability compared to non-inducer pollen due to delayed pollen germination (Xu et al. 2013). Within the pollen grains of an inducer, Bylich and Chalyk (1996) noticed pairs of morphologically different sperm nuclei at high frequency (6.3%). It was proposed that such differences could arise from the different speeds at which the two sperm cells develop, leading to a state where one sperm is ready for fertilization and another is not. Aneuploid microsporocytes occur at high frequency in haploid inducers (Chalyk et al. 2003; Qiu et al. 2014; Li et al. 2017), which could be due to abnormal division of chromosomes leading to the development of aneuploid sperm.

There are two conflicting hypotheses propounded to explain the phenomenon of in vivo maternal haploid induction and experimental evidence is available to support both of them. The two hypotheses propose (i) single fertilization instead of normal double fertilization and (ii) elimination of paternal chromosomes after normal fertilization. The high frequency of heterofertilization and endosperm/embryo abortion when using inducer pollen indicates single fertilization instead of normal double fertilization. Microscopic analysis of embryogenesis after pollination with haploid inducer pollen revealed single fertilized ovules (Swapna and Sarkar 2012; Xu et al. 2013; Tian et al. 2018) providing further direct evidence for single fertilization. Together, these observations indicate that haploid induction by maternal haploid inducers results from anomalous fertilization where one sperm fertilizes the central cell but the other sperm cell does not fuse with the egg cell. However, the egg cell is stimulated to develop into a haploid embryo either by division of the central cell or an attenuated sperm cell.

Contrary to the above observations, there is also strong evidence for normal double fertilization when using the inducer pollen. Several studies have revealed the presence of inducer chromosome segments in maternal haploids and the doubled haploids derived from them (Fischer 2004; Zhang et al. 2008; Li et al. 2009; Zhao et al. 2013; Qiu et al. 2014), indicating the occurrence of double fertilization and subsequent elimination of inducer chromosomes from the developing embryo. When using an inducer with the *R1*-*nj* and high oil markers, haploids with embryos that are weakly pigmented with anthocyanins and embryos with high oil were detected, supporting the hypothesis that chromosomal fragments were integrated into the haploids (Li et al. 2009). When inducers equipped with cytogenetic markers like the B chromosome were used in induction crosses, B chromosomes were detected in haploids at a low frequency, providing direct evidence of selective chromosome elimination during haploid induction (Zhao et al. 2013). Another observation that supports double fertilization and chromosome elimination is occurrence of aneuploidy, mixoploidy, lagged chromosomes and micronuclei in mitotic cells of inducer-pollinated ovules and the developing embryo/endosperm, all of which indicate chromosomal loss during haploid induction (Wedzony et al. 2002; Chalyk et al. 2003; Zhang et al. 2008; Zhao et al. 2013; Qiu et al. 2014; Li et al. 2017). In a cross between an inducer with normal endosperm and a sweet corn line with shrunken endosperm, F1 kernels with mosaic endosperm consisting of normal endosperm and shrunken endosperm were observed, indicating the loss of inducer chromosomes (Zhang et al. 2008). It has also been found that inducer chromosomes are eliminated in haploid embryonic cells during the first week after pollination (Zhao et al. 2013). Overall, it appears that both single fertilization and chromosomal elimination after double fertilization could be involved in maternal haploid induction in maize.

#### Haploid inducer development and maintenance

The main objectives in haploid inducer breeding are improving HIR, integrating new markers for haploid identification, and improving agronomic performance. There may also be a need to develop new haploid inducers to suit different target environments. Studies have indicated that HIR can be improved through selection of transgressive segregants that show a higher HIR compared to the parents in crosses involving either two inducers (Röber et al. 2005) or an inducer and a non-inducer (Chaikam et al. 2018). To develop new haploid inducers, a breeding scheme was proposed based on the pedigree method, which involves selection of individual plants based on highly heritable traits in the F_2_ generation, followed by selection among the families for HIR (Prigge et al. 2012a). Backcrossing can also be used if the objective is to improve adaptation or agronomic traits without necessarily sacrificing HIR (Prigge et al. 2012a; Chaikam et al. 2018). Improvement in agronomic performance for many traits relevant to the tropics has been demonstrated through selection in both selfing and backcrossing families derived from inducer x non-inducer crosses (Prigge et al. 2012a; Chaikam et al. 2018). New marker traits can be integrated into haploid inducers by crossing inducers with germplasm that has genetic markers and selecting for the marker expression in early generations and selecting for the HIR in families fixed for marker expression.

One important task in inducer breeding is precise determination of the HIR in plants or families resulting from inducer x inducer crosses or inducer x non-inducer crosses. The *R1*-*nj* marker integrated in the inducer parent is not suitable for this purpose because it leads to high misclassification rates as described below. Also, in inducer x non-inducer crosses, the *R1*-*nj* marker will segregate in early generation plants/families making it unusable. Hence, accurate measurement of the HIR is not straightforward as any specific morphological traits present in the inducers cannot be used. Recently, it was demonstrated that the HIR could be accurately measured by crossing the putative inducer candidates with testers carrying recessive genes for leaf morphology (*lg2,* liguleless; *gl* glossy) or herbicide resistance (Melchinger et al. 2016a). In such crosses, only haploids express recessive phenotypes, thus facilitating visual discrimination of haploids from diploids. The *lg* phenotype has been extensively used in development of new inducers (Röber et al. 2005; Prigge et al. 2012a; Melchinger et al. 2016a). However, any tester that facilitates easy discrimination of haploids from diploids can be used because it was noted that the effects on haploid induction of the tester and inducer x tester interaction are small while the inducer effect on the HIR is highly significant (Melchinger et al. 2016a). Therefore, inducer ranking based on the HIR does not change depending on the tester. Nevertheless, enough testcross seeds need to be evaluated to be confident of the HIR measurement. Evaluation of at least 200 and 1000 test cross seeds is recommended for early generation families and advanced generations, respectively (Prigge et al. 2012a). Measurement of the HIR of putative candidate inducers in multiple cycles and different environments is also needed to confirm stability of the HIR, as several genes that affect the HIR may segregate, besides the fact that the environment can also influence the expression of haploid induction. Although the tester phenotypes offer accurate measurement of the HIR, generating the testcross seed, and planting and evaluating the phenotypes from many plants/families is resource intensive and limits the number of families that can be screened for their HIR.

MAS of the *qhir1* locus with significant positive effects on the HIR has been implemented for efficient development of new temperate and tropical haploid inducers (Dong et al. 2014; Chaikam et al. 2018). The MAS of *qhir1* is very useful for eliminating plants/families lacking *qhir1*, which typically have no or very low HIR (Dong et al. 2014; Chaikam et al. 2018). Because plants/families with *qhir1* are selected against due to segregation distortion (Barret et al. 2008; Prigge et al. 2012b), MAS for *qhir1* in early generations helps to enrich *qhir1* genotypes. MAS saves resources in evaluating the HIR for plants/families and provides an opportunity for increasing selection intensity. In recent work, Chaikam et al. (2018) showed that in addition to MAS, haploid induction-associated traits can also be used to eliminate families that have no or low HIRs and demonstrated that *qhir1* conditions endosperm and embryo abortion in addition to haploid induction. Almost all the ears from families without *qhir1* had no or extremely low abortion while families with *qhir1* always showed abortion. Compared to families without endosperm abortion, families with endosperm abortion showed significantly higher HIRs. Moreover, families with high HIRs had higher proportion of haploids in the selfing or backcross progeny compared to families with no or low HIRs. Among the three traits, only endosperm abortion can be easily scored with minimal resources and hence can be easily implemented with or without MAS. Although these traits can distinguish plants/families with haploid induction vs. those without haploid induction, they cannot distinguish families with different HIRs. Despite using MAS or endosperm abortion, putative families with *qhir1* or endosperm abortion still need to be evaluated for HIR using recessive testers to determine the lines with the highest HIR.

Maintenance of inducer lines also entails frequent testing of the HIR because natural selection acts strongly against this trait (Melchinger et al. 2016a). Multiple studies showed a selective disadvantage for haploid induction, resulting in segregation distortion (Barret et al. 2008; Dong et al. 2013; Nair et al. 2017; Prigge et al. 2012b; Xu et al. 2013). Contamination of inducer stocks can quickly lead to reduction in the HIR because pollen from contaminants has a selective advantage over pollen from inducers. When increasing the seed of inducer lines, plants that do not show inducer line characteristics should be rigorously discarded. In each seed increase, 20–50 inducer plants can be tagged and crossed to recessive testers and also selfed. Test cross seed can be evaluated to determine the HIR and the selfed ears from plants showing the highest HIR can be used for further line maintenance. This ensures that high HIR is maintained in the inducer lines. Maintenance breeding also requires selection of agronomic traits such as pollen production and plant vigor, besides resistance to important diseases relevant for the target agro-ecology. To avoid loss of vigor during multiplication of inducers, sib mating within rows of multiplication plots is used instead of selfing (Chaikam et al. 2012).

### Haploid identification

In vivo induced haploids can be distinguished from diploids at the seed, seedling or adult plant stage. As haploids occur at a frequency of ~ 10%, most seeds or seedlings resulting from induction crosses are diploids and are of no use for DH line production. Therefore, it is highly advantageous to identify haploids at the seed stage because this would substantially reduce the number of seedlings/plants to be handled at later stages in the DH line development pipelines, thereby resulting in significant cost savings. Haploids and diploids can be distinguished based on the expression of genetic markers or based on innate differences in haploids and diploids.

#### Haploid/diploid sorting based on phenotypic markers integrated in the haploid inducers

**Fig. 2:**
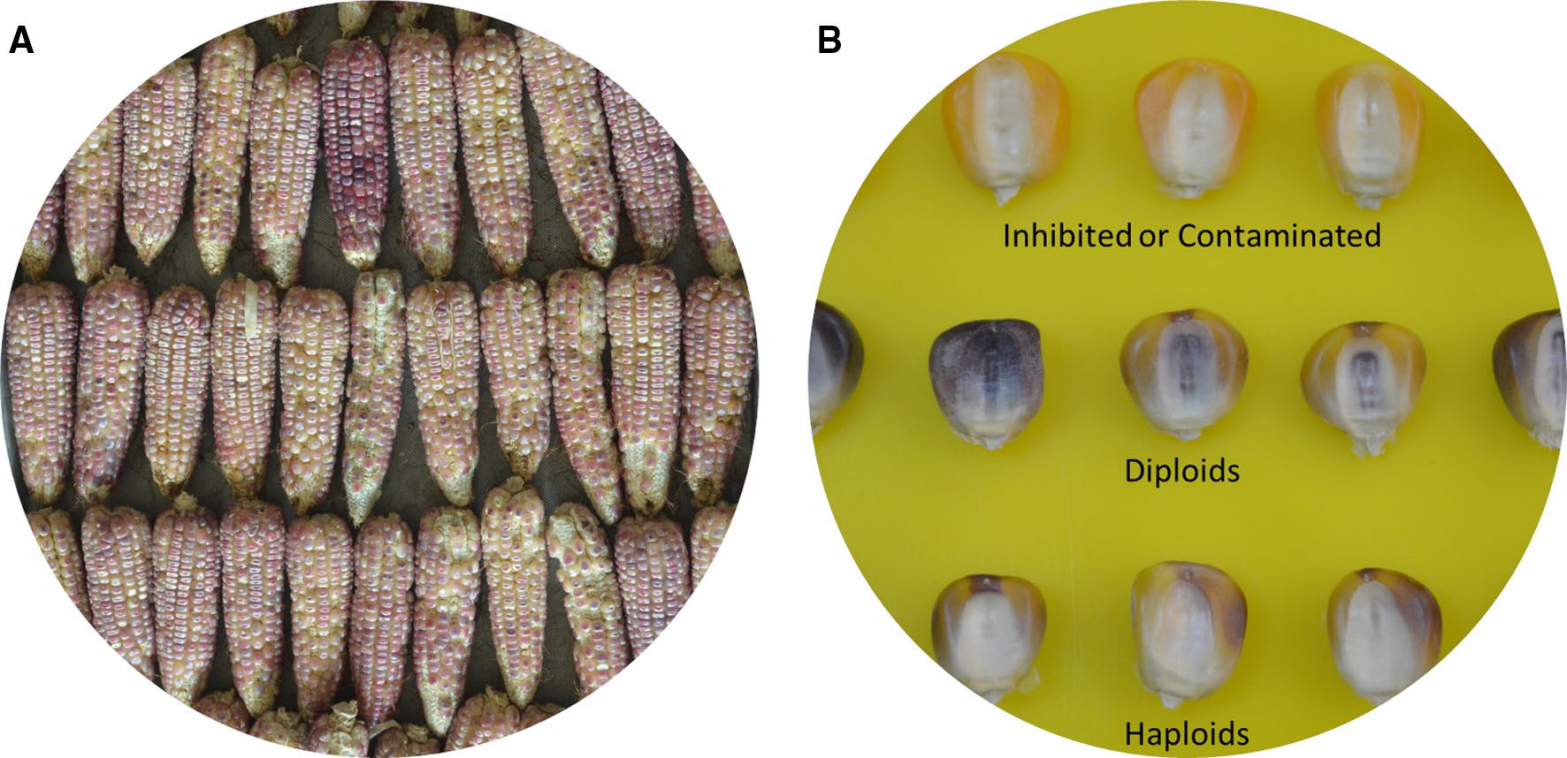
Haploid identification based on the *R1*-*nj* anthocyanin marker. **a** Ears from an induction cross showing *R1*-*nj* anthocyanin marker expression. **b** Classification of seed resulting from haploid induction cross into different categories

Genetic markers that are dominantly inherited and preferably expressed at the seed or seedling stage can be integrated into the maternal haploid inducers to aid in haploid identification. When such inducers are used in induction crosses, diploids are generally hybrids between the inducer (male parent) and the source germplasm (female parent), and thus have genome complements from both the male and female parents, while haploids only have the maternal chromosome complement. Genetic markers are especially important to identify haploids from diploids at the seed stage because haploid and diploid seeds look alike morphologically and cannot be separated based on visual inspection. The seed expressed anthocyanin color marker, *R1*-*nj* (Navajo), is widely used for haploid identification. All the currently used haploid inducers worldwide are equipped with *R1*-*nj* (Chaikam and Prasanna 2012; Melchinger et al. 2013). *R1*-*nj* in combination with other genes involved in the anthocyanin biosynthetic pathway, such as *A1, A2, C2, Bz1, Bz2* and *C1* conditions purple coloration in the aleurone layer of the endosperm and the scutellum of the embryo in diploids, which is commonly referred as the Navajo phenotype (Nanda and Chase 1966; Greenblatt and Bock 1967). Haploid seed resulting from induction crosses express anthocyanin only on the endosperm and not on the embryo, thus facilitating their differentiation from diploid seed on visual inspection (Nanda and Chase 1966) (Fig. 2). Recently, mechanical sorting has been optimized based on *R1*-*nj* marker expression using multispectral (De La Fuente et al. 2017), hyperspectral (Wang et al. 2018) and fluorescence imaging techniques (Boote et al. 2016), to reduce the time and labor involved in identification of haploids via the Navajo marker, but reliable estimates of misclassification by these methods are currently not available.

Although the *R1*-*nj* marker is most commonly used, its use in haploid identification suffers from some practical limitations. The most important limitation is that *R1*-*nj* marker expression can be inhibited due to dominant anthocyanin inhibitor genes like *C1*-*I*. It has been shown that *R1*-*nj* marker expression is inhibited in a significant proportion (~ 30%) of tropical elite inbred lines (Chaikam et al. 2015). In populations derived by crossing such inbred lines, it is not possible to identify haploids based on the Navajo phenotype. Such inhibition is also noted to be common in temperate flint germplasm (Röber et al. 2005). In addition, significant proportions of tropical breeding populations and landraces show segregation for *R1*-*nj* marker expression (Chaikam et al. 2015). In such germplasm, only a proportion of haploids present in the induction crosses can be identified. Molecular marker assays have been developed using sequence variation in the *C1*-*I* gene, which enable prediction of *R1*-*nj* marker inhibition or expression (Chaikam et al. 2015). These marker assays help conserve resources involved in haploid induction when dealing with populations with anthocyanin inhibitor genes. In addition to inhibition, the *R1*-*nj* marker can lead to high numbers of false positives (Röber et al. 2005; Prigge et al. 2011; Melchinger et al. 2014; Chaikam et al. 2016) and false negatives (Röber et al. 2005; Chaikam et al. 2016) resulting in wastage of resources in the downstream process. One more limitation of the *R1*-*nj* marker is the masking of the Navajo phenotype in germplasm that expresses purple or red anthocyanin coloration on the pericarp or on the outer layer of the endosperm. Many tropical maize landraces express such anthocyanin coloration on the seed, and consequently the *R1*-*nj* marker is not useful in such germplasm for haploid identification (Chaikam et al. 2016). Thus, there is a need for additional marker systems or methods to identify haploids accurately and efficiently in most maize germplasm.

A genetic marker that can aid haploid identification at the seed stage and can facilitate automation is based on high oil xenia effects. Normal maize germplasm contains about 3–4% oil, with > 85% of it accumulating in the embryo (Preciado-Ortiz et al. 2013). Haploid seeds and diploid seeds naturally show differences in their average kernel oil content with haploids showing 0.6–0.8% less oil than the diploids (Rotarenco et al. 2007; Melchinger et al. 2014). However, it was demonstrated that the haploids and diploids show a mixture distribution of oil content with a huge overlap and small differences in their mean oil content, making it impossible to separate haploids and diploids based on natural differences in oil content (Melchinger et al. 2013, 2014). Therefore, it is mandatory to use haploid inducers with an integrated high oil trait to reliably separate haploids and diploids based on oil content (Melchinger et al. 2014). In addition to automation, the high oil marker is not genotype dependent, making its use applicable in nearly all germplasm including landraces and wild relatives of maize like teosinte, with the exception of high oil maize germplasm. High oil inducers such as CAUHOI (7.8% Oil content (OC)) (Chen and Song 2003), CHOIL (~ 8.5% OC) (Dong et al. 2014) and UH600 (10.8% OC) and UH601 (11.7% OC) (Melchinger et al. 2013) were developed in temperate genetic backgrounds from crosses of maternal haploid inducers with high oil germplasm. When using such high oil inducers, the haploids showed lower mean oil content than diploid seed. However, the mean oil content of haploids and diploids depends on the oil content of the source germplasm and the inducer. The oil content of the inducer must be at least 10% to warrant a clear separation of both fractions because otherwise the proportion of false positives and false negatives is very large. Based on these observations, a criterion for controlling misclassification rates was developed for discrimination of haploid and diploid seeds obtained using high oil inducers (Melchinger et al. 2014). Completely automated high throughput platforms were recently described for sorting of single seeds via nuclear magnetic resonance to separate haploid seeds from diploid seeds based on oil mass (Wang et al. 2016) or oil content (Melchinger et al. 2017, 2018). The latter platform showed very high repeatability for kernel oil content measurements and high accuracy of classifying haploids and diploids, indicating a promising future for automation of haploid identification process. The limitations of using high oil markers are the availability of high oil inducers and the initial cost of establishing the NMR-based automated platform.

At the seedling stage haploids and diploids can be separated based on the expression of the red/purple root color marker and inducers that combine *R1*-*nj* and red root markers (Rotarenco et al. 2010; Chaikam et al. 2016). When using such inducers for haploid induction crosses, diploid seedlings express red/purple coloration in roots while haploids express no anthocyanin coloration, thereby facilitating easy separation of both classes. The advantages of the red/purple root color marker are that red root phenotype is very rare in maize germplasm and expressed independently of the *R1*-*nj* marker (Chaikam et al. 2016). Hence, it can be used in a wide array of maize germplasm. However, use of the red root marker for haploid identification would demand germination of large numbers of induced seeds, which is labor intensive. Therefore, use of the red root marker is recommended when the *R1*-*nj* marker is not effective due to complete or partial inhibition or when *R1*-*nj* marker expression is masked by natural anthocyanin expression in the seed. The red root marker can also be effectively used to reduce the false positives among haploids identified with the *R1*-*nj* marker.

The purple sheath marker has been integrated into several haploid inducers (Röber et al. 2005; Li et al. 2009; Prigge et al. 2012a) and was proposed as a way to eliminate false positives in the haploid fraction due to the *R1*-*nj* marker. However, the purple sheath marker was shown to be expressed naturally at a high frequency of ~ 60% in tropical landraces and ~ 10% in elite CIMMYT inbreds (Chaikam et al. 2016). In addition, this marker cannot identify haploids before chromosomal doubling treatments because it is only expressed at later stages of plant establishment (Chaikam et al. 2016). As a result, this marker is of little use in haploid identification or in timely elimination of false positives.

Attempts have also been made to identify haploids at the seed or seedling stage based on transgenic markers integrated into the inducers. The Engineered Green Fluorescent Protein (EGFP) marker with expression driven by the 35S promoter was integrated into a temperate haploid inducer (Yu and Birchler 2016). In the induction crosses, EGFP was noted as being expressed in the endosperm, embryo, roots and coleoptile of emerging diploid seedlings. For this reason, EGFP can be used at both the pre-emergence and post-emergence stages but not at the dry seed stage. It can also be used in embryo rescue procedures. Even though the haploids resulting from selection with transgenic inducers are generally free of transgenes, the application of transgenic haploid inducers may not be possible in many countries due to restrictions on the use of transgenes.

#### Haploid/diploid sorting based on innate differences between haploids and diploids

Haploids show differences in several characteristics compared to diploids owing to the reduced chromosome number, which can be exploited to distinguish them from diploids. Studies have indicated that haploids and diploids have different seed weights (Melchinger et al. 2014; Smelser et al. 2015), but the weight distributions of haploid and diploid seeds that result from induction crosses overlap to a large degree (Melchinger et al. 2014). So, it is not reliable to separate seed from an induction cross on the basis of seed weight alone. Seedling traits like coleoptile length, radicle length, and number of seminal roots also differ significantly between diploids and haploids with diploids having significantly higher values for all the traits (Chaikam et al. 2017). However, separating haploids and diploids based on seedling traits requires planting and evaluating large numbers of induced seed, which can be resource intensive. It is therefore recommended to use seedling traits only when no other marker systems are applicable. Nevertheless, use of seedling traits was shown to be highly effective to reduce false positives resulting from the use of *R1*-*nj* marker (Chaikam et al. 2017) and potentially can be used in combination with any other marker.

Mean stomatal size has also been noted to vary significantly between haploid and diploid seedlings and adult plants and was proposed as a method to differentiate haploids and diploids (Choe et al. 2012). However, the variance in the mean stomata lengths of individual plants is large, which makes reliable sorting difficult and only recommendable when no other marker system is available and image analysis for stomata measurement is at least partially automated (Molenaar et al. 2019a). Flow cytometry was only effective in distinguishing diploid from haploid seedlings in plant material that was not treated with chromosome doubling agents and furthermore carries the disadvantage of requiring expensive equipment (Molenaar et al. 2019a).

During vegetative and reproductive growth stages, maize haploid and diploid plants show very clear differences in many aspects. Haploid plants show distinctly poor vigor, erect and pale leaves with smaller leaf width, and no or poor pollen and seed production (Chase 1964b, 1969; Liu et al. 2017a, b; Wu et al. 2017). However, identification of haploids at the vegetative or reproductive stages with these characteristics is very inefficient and costly because it is highly resource-intensive. Thus it is not recommended for large-scale DH line production.

In conclusion, haploid inducers with a combination of phenotypic markers can be developed and deployed for reliable haploid identification in diverse germplasm. For example, when an inducer equipped with high kernel oil, *R1*-*nj* and red root markers is used, most diploids can be discarded based on oil content in a high throughput manner, followed by reconfirmation of putative haploids on the basis of *R1*-*nj* and red root markers. Such inducers with multiple genetic markers will increase the efficiency and accuracy of haploid identification.

### Chromosomal doubling

Haploids are generally sterile (Chaikam and Mahuku 2012) because meiotic divisions cannot occur, and this results in non-formation of gametes. Indeed, the chromosomes need to be doubled so that homologous chromosomes can pair, and meiosis continues normally, resulting in restoration of fertility. However, a high proportion of haploids (~ 97–100%) produce seeds when pollinated with pollen from normal diploid plants (Chalyk 1994; Geiger et al. 2006), whereas most haploids are male sterile. Consequently, restoring haploid male fertility (HMF) is generally considered as a limiting factor in production of DH lines (Chalyk 1994; Kleiber et al. 2012; Ren et al. 2017a; Wu et al. 2017). Restoring fertility in haploids can be achieved by artificial chromosomal doubling methods that rely on certain chemicals or spontaneous chromosomal doubling that relies on the innate ability of maize haploids to become spontaneously fertile.

#### Artificial chromosomal doubling

**Fig. 3:**
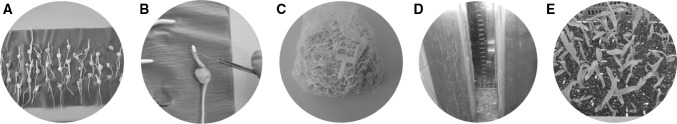
Artificial chromosomal doubling in putative haploids. **a** Germination of putative haploid seed (*D*_0_ seed) on paper towels. **b** Cutting of the coleoptile tip of *D*_0_ seedlings to facilitate better penetration of colchicine. **c** Placement of coleoptile cut *D*_0_ seedlings into mesh bags. **d** Treatment of *D*_0_ seedlings with colchicine in an iron tank. **e** Recovery of treated *D*_0_ seedlings in a greenhouse

Artificial chromosomal doubling is achieved by treating the haploid seedlings with chemicals that exhibit anti-mitotic activity. Colchicine is widely used for chromosomal doubling in DH line production pipelines (Chaikam and Mahuku 2012; Melchinger et al. 2016b). Colchicine binds to β-tubulin and prevents the formation of tubulin dimers thereby preventing formation of microtubules. Lack of microtubules during mitosis in the meristematic cells of the shoot apex prevents separation of replicated chromosomes, polar migration and cell division, resulting in a cell with a doubled chromosome number. The standard protocols involve immersing seedlings that have about 2 cm long coleoptiles in colchicine (0.04–0.06%) solution with 0.5% Dimethyl Sulfoxide (DMSO) for 8–12 h (Chaikam and Mahuku 2012; Prigge and Melchinger 2012) (Fig. 3). These protocols are referred to as seedling immersion methods and were described in the 1990s (Gayen et al. 1994; Deimling et al. 1997). In these protocols, haploid seeds are germinated on paper towels for 96–120 h until the coleoptiles are about 2 cm long. The coleoptile tip is cut off before submergence in the treatment solution to facilitate uptake of the doubling chemicals. Subsequently, seedlings are washed under tap water, planted in peat moss in trays or biodegradable pots in a greenhouse for recovery until the three-leaf stage (10–15 days depending on the growth of the haploids) and then transplanted to a DH nursery field (Prasanna 2012; Prigge and Melchinger 2012). In this method, a success rate of 10–30% can be obtained depending on the population (Chaikam and Mahuku 2012; Melchinger et al. 2016b). However, colchicine is toxic and needs to be handled and disposed of carefully (Chaikam and Mahuku 2012; Melchinger et al. 2016b).

Thus, replacing colchicine with less toxic alternatives in chromosomal doubling protocols is desirable (Chaikam and Mahuku 2012). Recently, chromosomal doubling processes based on anti-mitotic herbicides and nitrous oxide (N_2_O) gas have been optimized. Several herbicides inhibit microtubule assembly or microtubule organization and could be anti-mitotic (https://hracglobal.com/tools/world-of-herbicides-map). Melchinger et al. (2016b) demonstrated that a chemical mixture of 0.5% dimethyl sulfoxide, 20 mg L^−1^ Amiprophos-methyl (APM) and 4 mg L^−1^ pronamide applied to the seedlings as described above for standard colchicine treatment could result in an overall success rate of chromosomal doubling close to those achieved with colchicine. Amiprophos-methyl and pronamide are several hundred orders of magnitude less toxic than colchicine, based on oral LD50 values (Melchinger et al. 2016b). The anti-microtubule herbicides oryzalin, trifluralin and flufenacet were tested for in vitro DH production (Wan et al. 1991) and in vivo DH production (Melchinger et al. 2016b), but, the results were not promising in the concentrations and combinations applied. Although anti-mitotic herbicides show slightly lower efficiency than the colchicine-based protocols, their use may minimize the health risks to people conducting doubling treatments and may reduce the costs of chromosomal doubling because they are less toxic and do not need to follow such strict disposal procedures.

Another alternative to colchicine treatment developed by Molenaar et al. (2018) employs N_2_O gas. N_2_O inhibits polymerization of microtubules (Kitamura et al. 2009). Nitrous oxide gas, which is used as an inhalation anesthetic in dentistry and as a food packaging gas, is relatively safe or has essentially no negative health effects. The treatment involves seedlings of the same size as in standard colchicine treatment, which are placed in a pressure chamber with calcium hydroxide to absorb carbon dioxide from respiring seedlings. N_2_O gas is pumped into the chamber at a pressure of 0.6 MPa and seedlings are treated at 25 °C for 3 days. After treatment, the seedlings are further handled as described for colchicine and herbicide treatments. In comparison with colchicine and herbicide treatment, N_2_O treatment is less dependent on specialized facilities because N_2_O does not require special disposal and may be released into the atmosphere in a well-ventilated area. N_2_O has shown success rates similar to colchicine. However, this treatment requires investment in a pressure chamber that can withstand high pressures, and this may increase the cost of initial establishment (Kato 2002; Molenaar et al. 2018).

Besides the standard seedling immersion method for in vivo DH production, other treatment methods like application of colchicine to seeds (Gayen et al. 1994), and injection of colchicine into the shoot apical meristem (Zabirova et al. 1996) have been described. Application of 0.06% colchicine at 18 °C to the seeds with a small portion of plumule cut off resulted in higher chromosomal doubling rates than seedling dip treatments in experiments by Gayen et al. (1994), but similar experiments by Chalyk (2000) resulted in no fertile plants. At CIMMYT, application of colchicine at various concentrations for 5 h to seeds immersed in water for 18 h has not resulted in any significant differences in chromosomal doubling rates compared to spontaneous chromosomal doubling rates (unpublished data). Injection of colchicine solution 2–3 mm above the shoot apical meristem results in chromosomal doubling but the proportion of fertile plants was less than the standard seedling dip method (Chalyk 2000). Treatment of 10–12 days old seedling roots was evaluated by Deimling et al. (1997) for colchicine and Melchinger et al. (2016b) for anti-mitotic herbicides but proved to be much less efficient in doubling the haploid genomes compared to seedling immersion treatments. N_2_O treatment of adult maize plants at the floral primordial stage (growth stages V3-V8) with 0.6 MPa was published by Kato (2002) and subsequently patented (Kato 2006). Although the method showed promise in doubling the haploid genome, it is not high throughput because treatment of adult plants at the flowering stage requires bigger chambers and the plants need to be grown in pots. Another method of in vivo chromosome doubling is spraying of plants grown in the field; however, there are no reports published on this method yet. This method can potentially reduce the labor costs involved in potting and transplanting treated seedlings, which accounts for a large portion of the costs in standard seedling immersion treatment (Melchinger et al. 2016b).

#### Spontaneous chromosomal doubling

Another alternative to chemical-induced chromosomal doubling is to rely upon spontaneous chromosomal doubling and spontaneous restoration of haploid fertility where haploid plants produce pollen and seed without chemical treatment. This phenomenon was first described by Chase (1949b). The rate of spontaneous chromosome doubling may be calculated as the proportion of fertile plants (FP) that set seed after self-pollination within all the haploid plants (Kleiber et al. 2012), and this value is similar to the overall success rate described in the previous section for artificial chromosome doubling. FP has been studied in different maize germplasm groups and ranged from 0 to 16.7% in intra-pool crosses from Stiff Stalk, Lancaster, and Iodent heterotic groups (Kleiber et al. 2012). Elite tropical germplasm showed a significantly higher FP than tropical landraces, although both types of germplasm had mean rates of ≤ 1% (Kleiber et al. 2012). Anther emergence rate (AER), defined as the proportion of plants with emerged anthers, has been proposed as an effective measure of male fertility (Kleiber et al. 2012; Ren et al. 2017a; Wu et al. 2017). In diverse US and Chinese germplasm, the AER ranged from 9.8 to 89.8% with significant genetic variance (Wu et al. 2017).

In most maize germplasm, the rate of spontaneous chromosomal doubling is too low for DH production (Kleiber et al. 2012). However, recent studies indicate huge genetic variation and high heritability for spontaneous doubling and fertility restoration so that improvement by selection should be promising (Kleiber et al. 2012; Ren et al. 2017a; Wu et al. 2017; Ma et al. 2018). At CIMMYT, study of spontaneous fertility restoration in haploids derived from 330 tropical inbred lines revealed substantial genetic variation and high heritability for haploid fertility traits (Chaikam et al. 2019). A few of these tropical inbred lines have shown an extraordinary capacity for spontaneous chromosomal doubling and have fertility restoration rates that are superior to success rates achieved using artificial chromosomal doubling. These lines can be used as donors to improve spontaneous fertility restoration in tropical maize germplasm. The possibility of improving spontaneous doubling through selection was verified by Molenaar et al. (2019b) and showed that with three cycles of recurrent selection for HMF, the spontaneous chromosomal doubling rate could be increased from ~ 5% to 50% in two populations. Further, the authors found a predominance of additive effects, but also epistatic effects for spontaneous chromosomal doubling.

Since HMF is a limitation for DH line production through spontaneous doubling, it has been the focus of recent genetic studies. A study by Wu et al. (2017) revealed that HMF is controlled by two or more major genes with additive effects. Mapping studies using biparental mapping populations derived from parents with low and high HMF revealed several QTLs affecting HMF (Ren et al. 2017b; Yang et al. 2019).

The causes behind spontaneous chromosome doubling are still elusive, but somatic cell fusion, endoreduplication, endomitosis and nuclear restitution have been proposed as possible mechanisms (Jensen 1974; Shamina and Shatskaya 2011; Testillano et al. 2004). Spontaneous chromosome doubling commonly results in chimeric plants, which are comprised of a mosaic of both haploid and diploid cells. As a result, usually only small sectors of a tassel become fertile with pollen shedding anthers (Kleiber et al. 2012). Hence, it is common to see poor seed set resulting from spontaneously doubled haploids compared to haploids subjected to artificial chromosomal doubling. However, Molenaar et al. (2019b) observed an improvement in seed set by recurrent selection for spontaneous chromosome doubling, presumably due to improved pollen shed.

### Seed production from doubled haploid lines in the *D*_0_ nursery

**Fig. 4:**
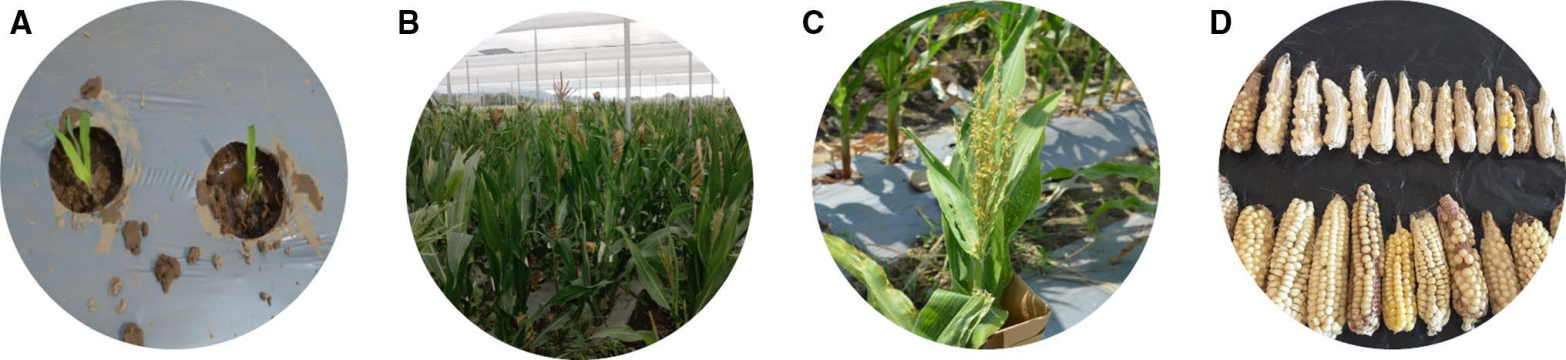
Agronomic management of *D*_0_ plants and seed production for DH lines. **a** Transplanted *D*_0_ seedlings in the field with plastic mulch. **b***D*_0_ plants under optimal growing conditions. **c** A fertile *D*_0_ plant that was self-pollinated. **d** Representative ears obtained from colchicine treated *D*_0_ plants from a single source population

Haploid plants are generally weak and show susceptibility to various biotic and abiotic stresses (Mahuku 2012). Chromosomal doubling treatments impose further stress, thereby increasing the mortality of seedlings in the greenhouse and/or in the field. Haploids therefore need to be handled with care during and after chromosomal doubling treatments, and while transplanting and growing in the field till harvesting the ears (Fig. 4). Improved irrigation methods like drip irrigation or sprinkler irrigation could help to avoid any water stress. In environments that are warm with high intensity sunlight, shade nets can reduce heat and light exposure. A regimented crop protection program needs to be implemented to avoid possible damage by insect pests and diseases. Weeds need to be controlled, especially at the early stages of crop growth. Haploids show sensitivity to post-emergent herbicides at rates applied to normal maize crops, so plastic mulch may be used to reduce weeds around haploid plants. In addition, application of foliar and soil fertilizers helps haploid plants to produce higher seed set on ears derived from chromosome doubled haploid plants.

As haploid tassels exhibit a wide variation in fertility that ranges from one or few anthers producing pollen to the whole tassel becoming fertile, haploid tassels need to be inspected regularly for any extruded anthers. Any tassels with extruded and plump anthers can be covered with wax-coated glassine tassel bags so that pollen is easily visible. Each *D*_0_ plant with fertile tassels can be self-pollinated two or three times on consecutive days to ensure good seed set. The ears with seeds can be harvested from *D*_0_ plants at physiological maturity. These seeds on each of the harvested ears represent a completely homozygous DH line, generally referred to as the D1 generation. The number of seeds obtained per D_1_ ear varies widely. Kleiber et al. (2012) reported an average of 4 seeds per D_1_ ear. At CIMMYT, depending on the season, 40–60% of the DH lines produced have more than 25 seeds whereas the rest have poor seed set (not published). The seed of DH lines can be directly planted for per se evaluations and for producing testcross seed or can be used for molecular marker applications.

## Future perspectives

Increased efficiency of DH line production and reduced cost per DH line will drive seamless integration of DH technology into maize breeding programs in the coming decades. Increased efficiencies can be achieved at the haploid induction stage by developing haploid inducers with high HIR and suitable agronomic performance for specific agroclimatic conditions. MAS of major loci critical for conditioning haploid induction and further phenotypic selection may enable development of new inducers with very high HIR (possibly > 20%). Automation of the haploid identification process using different phenotypic markers like high oil or *R1*-*nj* is becoming a reality with prototypes being developed by several research groups. In the future, haploid inducers equipped with multiple marker systems (e.g., high oil, *R1*-*nj* and red root) could be deployed in the DH process enabling high throughput and fool-proof haploid identification, thereby significantly reducing the costs incurred in haploid identification and managing false positives. Optimization of chromosomal doubling protocols based on spontaneous chromosomal doubling and/or non-hazardous chemicals is another important research area that could further increase the overall DH production efficiency. Optimization of growing conditions for *D*_0_ plants can improve the quantity and quality of seed of DH lines, which may eliminate the need to increase the seed of DH (D1) lines, thereby saving seed multiplication times and their associated costs. Use of embryo rescue protocols can significantly shorten the time (up to three months) in DH line development and can increase the rates of chromosomal doubling as most meristematic cells in the embryo can be exposed to doubling chemicals (Barton et al. 2008). Embryo rescue will be an integral part of DH production pipelines once the protocols for haploid embryo identification, chromosomal doubling and recovery of seedlings from embryos become widely available.

Thus, there are numerous opportunities for further refining the DH line production process and improving its efficiency. This can lead to novel applications for DH lines in maize breeding. For example, until recently DH technology was exclusively used for recycling elite germplasm to develop inbred lines for hybrid maize breeding. More recently, DH technology is being used for exploring the genetic diversity in maize landraces, for safeguarding genetic resources and introducing novel variation to expand the genetic base of elite germplasm (Melchinger et al. 2018, Böhm et al. 2017; Brauner et al. 2019). In addition, DH lines from exotic germplasm are being used to understand the genetics of adaptation-related traits (Vanous et al. 2018). Haploid induction-based genome editing (HI-Edit) or Haploid Inducer Mediated Genome Editing (IMGE) is a recent application that enables direct genomic modification of commercial inbred lines and eliminates several costly and time-consuming steps when incorporating genome-edited traits into elite cultivars (Kelliher et al. 2019; Wang et al. 2019).

Technological advances in maize DH production process are also propelling the evolution of efficient DH production methods in other plant species that are not amenable to in vivo haploid induction. For example, genome editing of the native rice matrilineal gene (OsMATL) resulted in in vivo haploid induction at a frequency of 2–6% (Yao et al. 2018). Use of PsASGR-BBML transgenes from wild apomictic grass species has been demonstrated to induce haploids in rice (Conner et al. 2017). Haploid induction systems based on engineered centromere specific histone H3 protein variant (CENH3) was demonstrated in Arabidopsis (Ravi and Chan 2010; Ravi et al. 2014) and maize (Kelliher et al. 2016) and may be extendable for many crop species. Extensive search for genotypes with haploid induction trait among 4000 germplasm entries in sorghum resulted in identification of haploid inducers with 1–2% HIR (Hussain and Franks 2019).

In addition to in vivo haploid induction, robust haploid identification methods applicable across plant species are also being conceived and tested. For example, use of transgenic double-fluorescence proteins comprising of eGFP driven by an embryo-specific marker and red florescent protein, dsRED, driven by an endosperm-specific marker, in conjunction with genome editing for the MTL gene, has been proposed as a potential strategy to enable haploid induction and robust haploid identification in many crop species (Dong et al. 2018). Together, with technological advances like the ones described in this review, the full potential of DH technology will be realized in the coming years in maize as well as in several other crop species.

## Abbreviations

DH: Doubled haploid
CIMMYT: International Maize and Wheat Improvement Center
MAS: Marker-assisted selection
QTL: Quantitative trait loci
OPVs: Open pollinated varieties
HIR: Haploid induction rate
TAILs: Tropically Adapted Inducer Lines
GWAS: Genome-wide association study
MTL: Matrilineal
EGFP: Engineered Green Fluorescent Protein
N_2_O: Nitrous oxide
FP: Fertile plants
AER: Anther emergence rate
HMF: Haploid male fertility
